# In vivo effects of AZD4547, a novel fibroblast growth factor receptor inhibitor, in a mouse model of endometriosis

**DOI:** 10.1002/prp2.759

**Published:** 2021-04-03

**Authors:** Sara Santorelli, Deborah P. Fischer, Michael K. Harte, Johanna Laru, Kay M. Marshall

**Affiliations:** ^1^ NorthWest Centre for Advanced Drug Delivery (NoWCADD) School of Health Sciences Faculty of Biology, Medicine and Health University of Manchester Manchester Academic Health Science Centre Manchester UK; ^2^ AstraZeneca Cambridge UK; ^3^ Division of Pharmacy and Optometry University of Manchester Manchester UK; ^4^ Early Product Development Pharmaceutical Sciences iMED Biotech Unit AstraZeneca Macclesfield UK

**Keywords:** animal model, endometriosis, estrogen, fibroblast growth factor receptor, progestin

## Abstract

Endometriosis is a chronic disease, characterized by the growth of endometrial‐like cells outside the uterine cavity. Due to its complex pathophysiology, a totally resolving cure is yet to be found. The aim of this study was to compare the therapeutic efficacy of AZD4547, a novel fibroblast growth factor receptor inhibitor (FGFRI), with a well‐characterized progestin, etonogestrel (ENG) using a validated in vivo mouse model of endometriosis. Endometriosis was induced by transplanting uterine fragments from donor mice in proestrus into the peritoneal cavity of recipient mice, which then developed into cyst‐like lesions. AZD4547 and ENG were administered systemically either from the day of endometriosis induction or 2‐weeks post‐surgery. After 20 days of treatment, the lesions were harvested; their size and weight were measured and analyzed histologically or by qRT‐PCR. Stage of estrous cycle was monitored throughout. Compared to vehicle, AZD4547 (25 mg/kg) was most effective in counteracting lesion growth when treating from day of surgery and 2 weeks after; ENG (0.8 mg/kg) was similarly effective in reducing lesion growth but only when administered from day of surgery. Each downregulated FGFR gene expression (*p* < 0.05). AZD4547 at all doses and ENG (0.008 mg/kg) caused no disturbance to the estrous cycle. ENG at 0.08 and 0.8 mg/kg was associated with partial or complete estrous cycle disruption and hyperemia of the uteri. AZD4547 and ENG both attenuated endometriotic lesion size, but only AZD4547 did not disrupt the estrous cycle, suggesting that targeting of FGFR is worthy of further investigation as a novel treatment for endometriosis.

## Graphical abstract

Fibroblast growth factor receptors (FGFRs) mediate metabolic functions, tissue development, repair, and regeneration, leading to endometriotic lesion growth and progression. Targeting this aberrant pathway may present a new therapeutic opportunity in endometriosis.

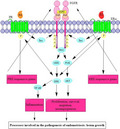

**Abbreviations**
af: atrophic follicle
cl: corpus luteum
E2: estradiol
ENG: etonogestrel
ERα: estrogen receptor alpha
FGFR: fibroblast growth factor receptor
FGFRI: fibroblast growth factor receptor inhibitor
FRS2: fibroblast growth factor receptor substrate 2
g: glandular structure with glandular epithelium
H&E: hematoxylin and eosin
HPSG: heparan sulfate proteoglycan
IKK/NF‐kB IκB: kinase/nuclear factor kappa‐light‐chain‐enhancer of activated B cells
l: leukocytes
L: lumen
le: luminal epithelium
MAPK: mitogen‐activated protein kinase
NSCLC: non‐small cell lung cancer
of: ovarian follicle
om: omental adhesion,
P4: progesterone
PDTX: patient‐derived tumor xenograft
PEG400: polyethylene glycol 400
PFA: paraformaldehyde
PI3K/AKT: phosphoinositide 3‐kinase/protein kinase B
PR: progesterone receptor
PRE and ERE: progesterone and estrogen responsive elements
pw: peritoneal wall
QD: once a day
s: stroma
S2: 2 weeks after surgery
SO: day of surgery

**Summary**

- What is already known:
  ▪ Progestins are used in endometriosis therapy.
- What this study adds:
  ▪ Inhibition of FGFR, through use of AZD4547, counteracts endometriosis development and progression without estrous cycle disruption.
- Clinical significance:
  ▪ FGFR inhibition could represent an effective target for endometriosis therapy without impairing fertility.

## 1 INTRODUCTION

**FIGURE 1:**
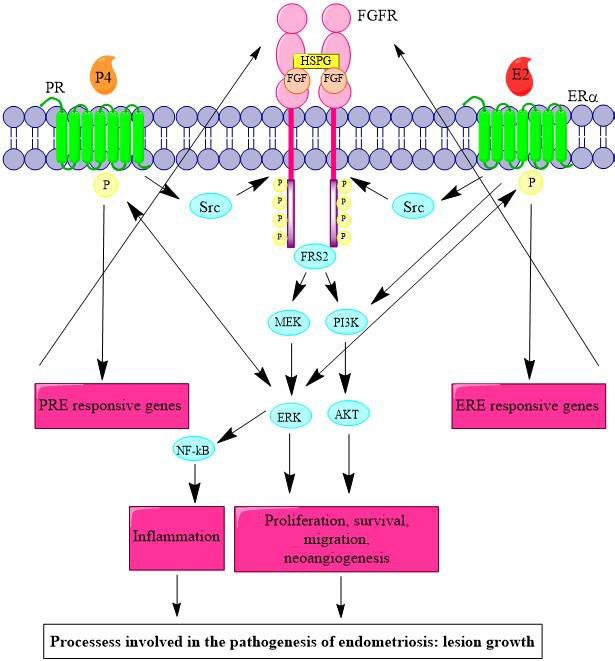
Progesterone (P4), estrogen (E2), and FGFR signaling pathways: P4 and E2 bind to their receptors PR and ERα. The binding to these receptors leads to the activation of Src kinases, which activates FGFR by phosphorylation (P). FGFR is a transmembrane receptor associated with heparan sulfate proteoglycan (HPSG), which stabilizes the FGF binding and prevents FGF from being degraded by protease enzymes. As consequence of the FGFR phosphorylation, fibroblast growth factor receptor substrate 2 (FRS2) activates the PI3K/ AKT, IκB kinase/nuclear factor kappa‐light‐chain‐enhancer of activated B cells (IKK/NF‐kB), and the MAPK signaling pathways, which can, in turn, activate ERα and PR receptors by phosphorylation. P4 and E2 can also activate transcriptional pathways, binding to specific DNA sequences (progesterone and estrogen responsive elements [PRE and ERE]) found on progesterone and estrogen responsive genes leading to activation of FGFR and lesion development in endometriosis^11^

Endometriosis is a condition with an estimated prevalence of 10%–15%,^1^ characterized by growth of endometrial‐like glands and stroma outside the uterine cavity.^2^ These ectopic endometrial cells respond to sex steroid hormone changes in the menstrual cycle, but their menstrual products cannot be cleared during menses. The repeated cyclical bleeding causes inflammation, chronic pelvic pain, and subfertility.^1^ Current therapies for endometriosis include treatments whose long‐term usage may induce side effects that limit their clinical usefulness.^3^ Suppressing estrogen signaling is central to current treatment strategies. However, more recently consideration that endometriotic lesions require nutrients and oxygen provided by blood vessels to sustain their growth and promote invasion and survival^4^ has sparked interest in anti‐angiogenic therapies as an alternative for endometriosis treatment.^5^, ^6^ Fibroblast growth factor receptor (FGFR) 1 was found to be overexpressed in the ectopic endometrium of women with endometriosis compared to the eutopic endometrium of women without and has been linked to endometriosis recurrence.^7^ High expression of FGFR2 is associated with risk of endometrioma carcinogenesis,^8^ and mutations are implicated in 12%–14% of endometrial cancer cases.^9^, ^10^ Activation of FGFRs phosphorylate estrogen receptor (ER) α and progesterone receptor (PR) via proto‐oncogene tyrosine‐protein (Src) kinases and phosphoinositide 3‐kinase/protein kinase B (PI3K/AKT) and mitogen‐activated protein kinase (MAPK) signaling pathways, leading to cell proliferation, survival, migration, and neovascularization (Figure 1).^7^ Inhibiting the FGFR cascade may offer much needed therapeutic opportunities for endometriosis.

AZD4547 is a selective inhibitor of FGFR1‐3, currently in clinical trials for several solid tumors.^10^ Based on its efficacy towards tumor regression, it was hypothesized that AZD4547 could be repositioned for endometriosis therapy. To investigate the therapeutic efficacy of AZD4547 on the establishment and development of lesions, a validated mouse model of endometriosis was used.^12^ Effects of administering AZD4547 from the day of endometriosis induction and 2‐weeks post‐surgery were chosen to evaluate its potential as a prophylactic or management therapy and compared with a characterized progestogenic agent, ENG (etonogestrel).^13^

## 2 MATERIALS AND METHODS

### 2.1 Test system

Non‐pregnant virgin female sexually mature C57/6 J mice (*n* = 124), aged 12–18 weeks, weighing 19–22 g (Envigo Ltd, UK), were housed in a room with constant controlled temperature (21 ± 2°C) and 45%–55% humidity with a 12/12‐h light/dark cycle and access to food and water ad libitum. The stage of the estrous cycle was assessed by daily vaginal lavage.^14^ Mice in proestrus were chosen as donors because the higher circulating E2 levels ensured the uteri were larger and more vascularised compared to other stages of the cycle.^15^, ^16^

### 2.2 Experimental design

The animal work was based on a well‐validated endometriosis mouse model^12^ and performed under the UK Home Office Project Licence Number (70/8458). All experimental procedures and animal care were conducted in accordance to the Animal (Scientific Procedures) Act 1986 Amendment Regulations (SI 2012/3039) and the Animal Welfare Act 2006. Test system and experimental design were reported in adherence to ARRIVE guidelines. Donor mice (*n* = 28) were humanely killed by cervical dislocation under Schedule 1 (S1). After harvesting the uteri, a longitudinal incision along each of the two horns was performed to open the lumen exposing the endometrial layer. These uteri were then cut into approximately 2 mm^2^ fragments, which were weighed. The recipient mice (*n* = 96) were anesthetized with 2% v/v oxygen isoflurane, their ventral area was shaved and cleaned with ethanol/betadine solution and 0.1 mg/ml of buprenorphine was administered. Body temperature and respiration were constantly monitored during the surgery, and Lacri‐lube ocular lubricant was used to avoid optical dryness. An incision on the right side of the peritoneal cavity of the mice was performed, and three uterine fragments were stitched onto the parietal peritoneum using Mersilk‐braided silk nonabsorbable sutures. Uterine fragments were attached unilaterally so that the endometrial layer was adjacent to the peritoneal wall of the recipient mice. Vicryl absorbable sutures were used to close the muscle layer underneath the incision and the skin, and betadine solution was applied to the wound. The mice were closely monitored after surgery, placed in an incubator with controlled temperature and easier access to food for an hour and subsequently housed in groups of six. Recipient mice were randomly divided into two main groups receiving once a day (QD) treatment with AZD4547 at the doses of 5 (*n* = 6), 12.5 (*n* = 6), 25 mg/kg (*n* = 6) and vehicle only (polyethylene glycol [PEG] 400) (*n* = 6) by oral gavage (p.o), or, ENG at the doses of 0.008 (*n* = 6), 0.08 (*n* = 6), and 0.8 mg/kg (*n* = 6) and vehicle only (sesame oil, *n* = 6) by subcutaneous injection (s.c.), starting from the day of endometriosis induction (S0, *n* = 48), or, 2 weeks after (S2, *n* = 48, when the lesions were already established),^12^ for 20 days. Dose ranges and length of treatments of AZD4547^10^, ^17^, ^18^ and ENG^19^ were chosen according to those published in the literature. AZD4547 was suitable for oral gavage due to its reported bioavailability, selectivity and anti‐tumor efficacy by this route, whereas ENG was administered parenterally to avoid its rapid metabolism by the liver. This also reflected their clinical routes of administration.

AZD4547 was prepared in a 30% v/v solution of PEG 400 in deionized water adjusted to pH 4. ENG was prepared in 5% v/v absolute ethanol and 95% v/v sesame oil. Twenty days after the start of the treatment, mice were killed by cervical dislocation under S1 to evaluate the therapeutic efficacy of AZD4547 and ENG towards the establishment and development of the endometriotic lesions. Lesions were weighed, and their volumes were measured using a caliper. Lesions were snap frozen or fixed overnight in 4% w/v paraformaldehyde (PFA) in phosphate‐buffered saline (PBS), paraffin embedded, sectioned, and stained with hematoxylin and eosin (H&E) to examine their structural changes. Blind testing was only possible when lesions were harvested but could not be performed when administering the freshly prepared drugs as different routes of administration were required (same operator).

### 2.3 RNA isolation and quantitative real‐time PCR

Total RNA was extracted from snap frozen lesions using TRIzol reagent and mirVana kits according to the manufacturers’ instructions. RNA quality was measured on a NanoDrop 2000 (Fisher Scientific, Loughborough, UK) before 1 µg was transcribed into cDNA using QuantiTect Reverse Transcription kits. Quantitative real‐time (qRT)‐PCR was performed on a Quantstudio 12 K flex real‐time PCR system (Applied Biosystems, Paisley, UK) using SYBR Green Supermix and primer sequences designed by our laboratory (NCBI Primer Blast; Table S1). Samples were run in triplicate using 0.5 µl cDNA in a total reaction volume of 10 µl. Gene expression was determined using the ΔΔCT method, normalized against β‐actin and GAPDH reference genes, and data were expressed as fold‐change relative to vehicle.

### 2.4 Data and statistical analysis

Data were analyzed using GraphPad Prism 8 and expressed as means ± standard error of the mean (SEM). Animal number per group *n* = 6, lesion number per group, *n* = 18, with the exception of the groups treated with the vehicle of AZD4547 and ENG at the dose of 0.8 mg/kg from 2 weeks after surgery, whose lesion numbers were respectively *n* = 15 and *n* = 17, due to the loss of the lesion fluid during the delicate process of excision of the cysts from the peritoneal wall and separation of the omentum. The group size was calculated using 80% power to detect 50% difference in lesion size at 5% significance level. Distribution of the data was analyzed using D'Agostino and Pearson Test. The significance between two dependent groups was evaluated using Wilcoxon test (nonparametric); the significance between independent groups was assessed using Student's *t* test (parametric) or Mann–Whitney test when comparing two groups (nonparametric) one‐way analysis of variance (ANOVA) or Kruskal–Wallis with Dunn's post hoc test for comparing two or more groups or two‐way ANOVA with Bonferroni post hoc test when two categorical variables were present. Post hoc tests were only conducted when *F* was significant and there was no variance in homogeneity. *p* values <0.05 were considered statistically significant: *p < 0.05, **p < 0.01, ***p < 0.001. The data and statistical analysis comply with the recommendations on experimental design and analysis in pharmacology.^20^

### 2.5 Materials

Lacri‐lube ocular lubricant was purchased from Allergan (CA, USA); Mersilk‐braided silk nonabsorbable sutures, and Vicryl absorbable sutures were supplied by Ethilon (Georgia, USA); Betadine was purchased from Meda Pharma (Solna, Sweden); AZD4547 (*N* [5‐ [2‐(3,5‐dimethoxyphenyl) ethyl]‐1*H*‐pyrazol‐3‐yl]‐4‐[(3*R*,5*S*)‐3,5‐dimethyl‐1‐piperazinyl]‐benzamide) was provided as a kind gift by AstraZeneca (Macclesfield, UK) and synthesized according to the processes described in the International Patent Application Publication Number WO2008/075068; PEG 400, ENG (3‐keto‐desogestrel), and TRIzol reagent were purchased from Sigma‐Aldrich (Leeds, UK); PFA, PBS, and the mirVana™ miRNA isolation Kit were supplied by Fisher Scientific (Loughborough, UK); Hematoxylin was supplied by Raymond A LAMB Ltd (Eastbourne, UK); eosin was purchased from Shandon, Thermoscience (Handforth, UK) and the QuantiTect Reverse Transcription kit and SYBR Green Supermix were from Qiagen (Manchester, UK).

## 3 RESULTS

### 3.1 AZD4547 administered from S0

**FIGURE 2:**
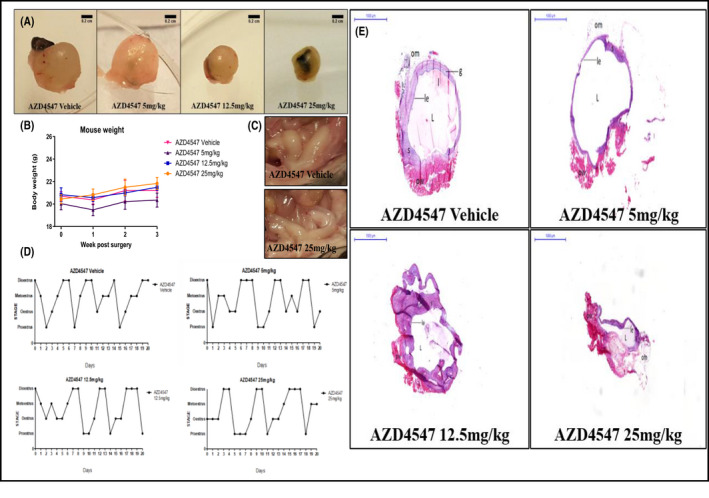
(A) Close ups of the lesions extracted from mice treated QD with vehicle (PEG400), AZD4547 at the doses of 5, 12.5, and 25 mg/kg p.o for 20 days from the day of endometriosis induction (the black nonabsorbable sutures used to attach the uterine fragments to the peritoneal wall were still visible). Scale bars are 0.2 cm. (B) Mice weight (g) fluctuations at Week 1, 2, and 3 post‐surgery and treatment. Data are expressed as mean ± SEM. (C) Close ups of the uteri extracted from mice treated with QD administration of vehicle and AZD4547 at the dose of 25 mg/kg p.o for 20 days from the day of endometriosis. (D) Typical estrous cycle of mice following endometriosis induction and QD vehicle and AZD4547 treatment p.o for 20 days. (E) Typical H&E staining of endometriotic lesions established in the mice‐treated QD with vehicle and AZD4547 p.o from the day of endometriosis induction for 20 days. Scale bars and magnification are respectively 1,000 μm and 1×. In the images: pw: peritoneal wall; L: lumen; om: omental adhesion; g: glandular structure with glandular epithelium; le: luminal epithelium; s: stroma; l: leukocytes

Following surgical induction of endometriosis, lesions developed in all groups, having the appearance of large cysts filled with fluid, clumped together, most with omental adhesions (Table S2). Stage of estrous cycle of recipients at the time of surgery (Figure S1) or sacrifice (Table S3; Figure S2) had no effect on final lesion size. In the AZD4547‐treated groups lesions appeared smaller than the vehicle group and not always filled with fluid (Figure 2A). Animals responded well to the treatment without any significant weight gain or loss, indicating tolerability of the drug (Figure 2B).

Uteri appeared well vascularized (Figure 2C; macroscopic differences between the groups, if present, were observed due to the stage of the estrous cycle [uteri taken at proestrus appeared more swollen compared to the others in other stages of the cycle]). Daily examination of vaginal cytology showed that the induction of endometriosis and treatment with AZD4547 administered from the day of surgery at every dose did not evoke estrous cycle disruption, as can be seen by the regularity of the four‐five days cycle in the vehicle and in the drug treatment groups (Figure 2D). H&E staining confirmed viable, well‐developed lesions with glandular and luminal epithelium, stroma with glandular structures, lumen filled with leukocytes in all groups. The groups treated with AZD4547 at the dose of 12.5 and 25 mg/kg showed reduction in lesion size compared to the vehicle and 5 mg/kg groups (Figure 2E).

**FIGURE 3:**
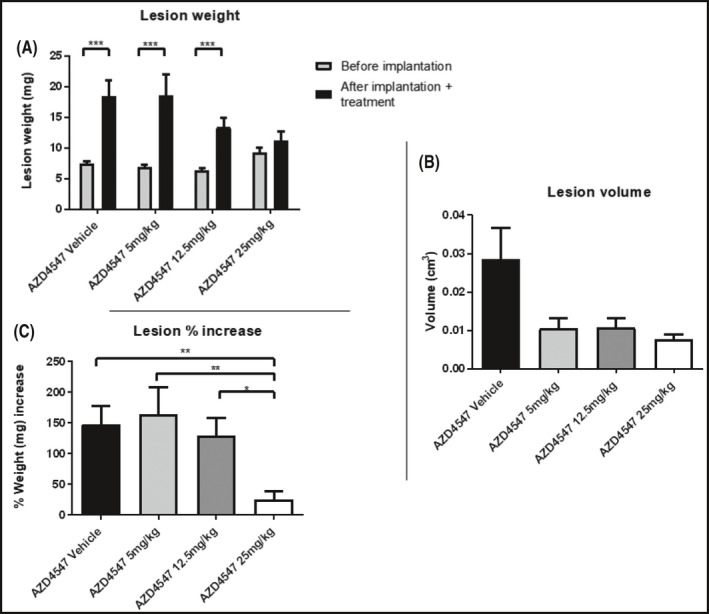
(A) Weight of the uterine fragments before being transplanted and after sacrifice following 20‐day treatment QD with vehicle, AZD4547 at the doses of 5, 12.5, and 25 mg/kg p.o starting from the day of surgery. (B) Final volume (cm^3^) of lesions with fluid. (C) Percentage increase in lesion weight. Data are expressed as mean ± SEM. Animal number per group *n* = 6, lesion number per group, *n* = 18

The increase in the weight of the lesions from the day of implantation to the day of harvest was statistically significant in the mice treated with the vehicle and AZD4547 at the doses of 5 and 12.5 mg/kg (***p < 0.001) but not in the 25 mg/kg group (Figure 3A **)**.

Lesion volume decreased for AZD4547‐treated groups compared to vehicle groups; this corresponded to increasing dose (Figure 3B). Compared to its vehicle, AZD4547 at 5 mg/kg was responsible for 63.3% volume reduction, 12.5 mg/kg for 62.5% and 25 mg/kg for 73%.

Lesion weight (expressed as a percentage increase in weight of the original fragment implanted, Figure 3C) was significantly higher in the AZD4547 vehicle group (**p < 0.01), in the groups treated with AZD4547 at 5 mg/kg (**p < 0.01) and 12.5 mg/kg (*p < 0.05) compared to the 25 mg/kg group. Compared to its vehicle, AZD4547 at the dose of 5 mg/kg was not responsible for lesion weight regression but for a 11.4% increase, whereas the doses of 12.5 and 25 mg/kg induced a regression of 12.2% and 83.3%, respectively. Percentage of lesions filled with fluid decreased passing from the vehicle (89%) to the drug‐treated groups (78%).

### 3.2 ENG administered from S0

**FIGURE 4:**
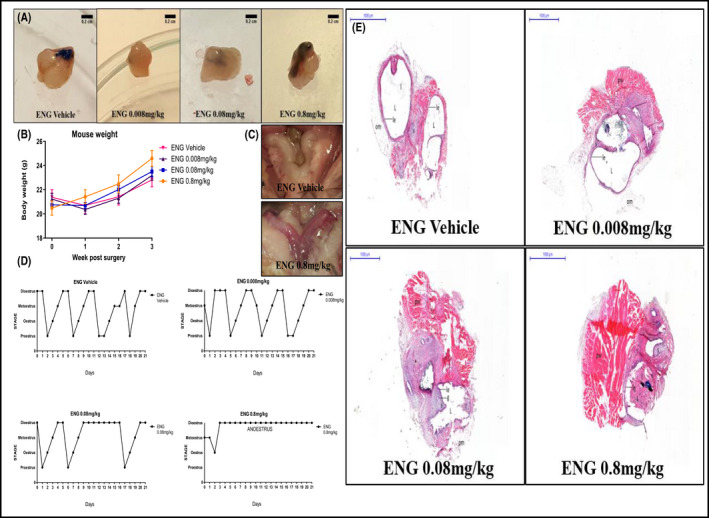
(A) Close ups of the lesions extracted from mice treated with QD administration of vehicle and ENG at the doses of 0.008, 0.08, and 0.8 mg/kg s.c. for 20 days from the day of endometriosis induction (the black nonabsorbable sutures used to attach the uterine fragments to the peritoneal wall were still visible). Scale bars are 0.2 cm. (B) Mice weight (g) fluctuations at Week 1, 2, and 3 post‐surgery and treatment. Data are expressed as mean ± SEM. (C) Close ups of the uteri extracted from mice treated with QD administration of vehicle and ENG at the dose of 0.8 mg/kg s.c. for 20 days from the day of endometriosis. The uterus from the mouse treated with the highest dose of the drug appeared hyperemic with vascular engorgement. (D) Typical estrous cycle of mice following endometriosis induction and QD treatment with vehicle and ENG s.c. from the day of endometriosis induction for 20 days. (E) Typical H&E staining of endometriotic lesions established in the mice treated QD with vehicle and ENG s.c. from the day of endometriosis induction for 20 days. Scale bars and magnification are respectively 1,000 μm and 1×. In the images: pw: peritoneal wall; L: lumen; om: omental adhesion; g: glandular structure with glandular epithelium; le: luminal epithelium; s: stroma; l: leukocytes

Following surgical induction of endometriosis, large cysts filled with fluid, clumped together with omental adhesion developed in the vehicle group. In the groups treated with ENG at 0.008 and 0.08 mg/kg, lesions appeared smaller than the vehicle group and not always filled with fluid, whereas in the 0.8 mg/kg group, no fluid‐filled cysts or lesion development was observed (Figure 4A). ENG was well tolerated, and no significant weight gain or loss was observed (Figure 4B); however, uteri appeared hyperemic with vascular engorgement increasing with dosage of the drug compared to the vehicle group (Figure 4C).

When ENG was administered from the day of surgery, the vehicle and the lowest dose (0.008 mg/kg) groups similarly showed no impairment of the 4‐5 day estrous cycle; interruption followed by recovery of the cycle regularity was observed in the 0.08 mg/kg treatment group, whereas the dose of 0.8 mg/kg caused complete disruption of the cycle and establishment of anestrus, with observation of mucus and thickening in the vaginal smear (Figure 4D). The lesions developed in the group treated with ENG at 0.008 mg/kg with similar histological appearance (glandular and luminal epithelium, stroma with glandular structures, a lumen filled with leukocytes) and size as seen in the vehicle group, whereas lumen reduction was shown in the 0.08 mg/kg group and no fluid‐filled lumen was observed in the 0.8 mg/kg group (Figure 4E).

**FIGURE 5:**
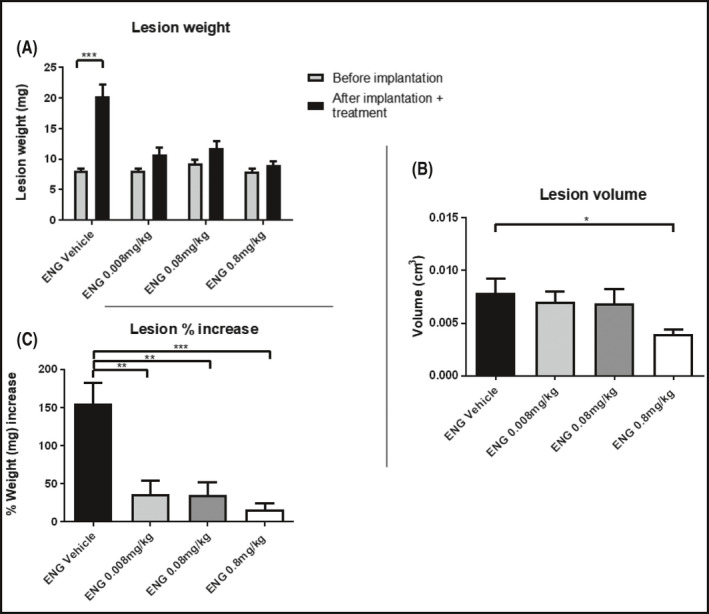
(A) Weight of the uterine fragments before being transplanted and after sacrifice following 20‐day QD treatment with vehicle, ENG at the doses of 0.008, 0.08, 0.8 mg/kg s.c. starting from the day of surgery. (B) Final volume (cm^3^) of lesions with fluid. (C) Percentage increase in lesion weight. Data are expressed as mean ± SEM. Animal number per group *n* = 6, lesion number per group, *n* = 18

The increase in weight of the lesions from the day of implantation to the day of harvest was statistically significant in the mice treated with vehicle (***p < 0.001) but not in any of the ENG treated groups (Figure 5A **)**.

Lesion volume decreased for ENG‐treated groups compared to the vehicle; this corresponded to increasing dose (*p < 0.05 when ENG vehicle was compared to the 0.8 mg/kg group, Figure 5B). Compared to its vehicle, ENG at 0.008 mg/kg reduced the volume by 10.6%, 0.08 mg/kg by 12.7% and 0.8 mg/kg by 50% (Table S4).

Lesion weight (expressed as a percentage increase in weight of the original fragment implanted, Figure 5C) was significantly higher in the ENG vehicle group compared to the 0.008, 0.08 (**p < 0.01), and 0.8 mg/kg treatment groups (***p < 0.001, Figure 5C). Compared to its vehicle, ENG at the dose of 0.008 mg/kg induced a lesion weight regression of 76.6%, the dose of 0.08 mg/kg of 77.1%, and the dose of 0.8 mg/kg of 89.2%. Percentage of lesions filled with fluid decreased from vehicle (94%) to the drug‐treated groups (50% in the 0.008 mg/kg group, 44% in the 0.08 mg/kg group; no fluid‐filled cysts development was observed in the group treated with ENG at the highest dose).

### 3.3 Downregulation of FGFR

**FIGURE 6:**
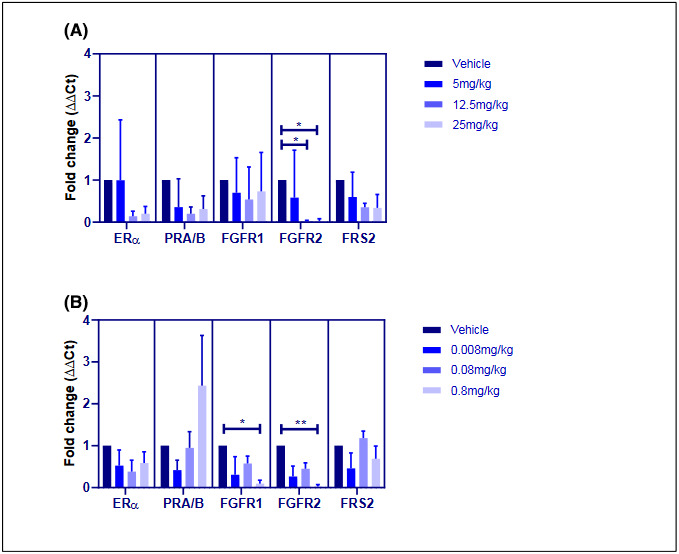
Expression of ERα, PRA/B, FGFR1, FGFR2 and FRS2 transcripts in lesions from mice treated with vehicle (A) AZD4547 at 5, 12.5, and 25 mg/kg or (B) ENG at 0.008, 0.08, and 0.8 mg/kg (*n* = 4/ group). AZD4547 and ENG were delivered QD by oral gavage or subcutaneous injection respectively for 20 days from the day of surgery. Total RNA was extracted using the TRIzol method, purified using a mirVana kit and quantified using qRT‐PCR. Gene expression was normalized against β‐actin and GAPDH and data were expressed as mean ± SEM relative to vehicle and analyzed using a Kruskal–Wallis test with Dunn's multiple comparisons post hoc adjustment; *p < 0.05 and **p < 0.01 compared to the vehicle

ERα was the most abundant gene transcript in lesions when mice were treated with vehicle for 20 days from the day of surgery. AZD4547 more than halved expression of ERα, PRA&B, and FRS2 compared to vehicle; however, these changes were not significant (Figure 6). While there was no effect on FGFR1, AZD4547 significantly reduced FGFR2 complements at 12.5 (*p* < 0.05) and 25 mg/kg (*p* < 0.05).

ENG similarly regulated gene transcription in mouse lesions. Expression of ERα and FRS2 was variable but lower than vehicle; conversely, ENG increased PRA/B mRNA by 2.4‐fold (*p* > 0.05). Only the reduction of FGFR1 (*p* < 0.05) and FGFR2 (*p* < 0.01) by ENG at 0.8 mg/kg were significant, where transcripts were barely detectable.

### 3.4 AZD4547 administered from S2

**FIGURE 7:**
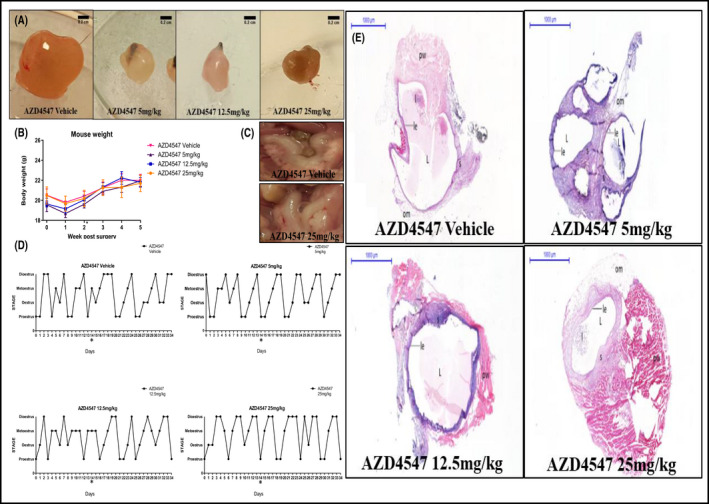
(A) Close ups of the lesions extracted from mice treated QD with administration of vehicle and AZD4547 at the doses of 5, 12.5, and 25 mg/kg p.o for 20 days from 2 weeks after endometriosis induction (the black nonabsorbable sutures used to attach the uterine fragments to the peritoneal wall were still visible). Scale bars are 0.2 cm. (B) Mice weight (g) fluctuations at Week 1, 2, 3, 4, and 5 post‐surgery and treatment. The symbol * by Week 2 indicates the beginning of the treatment. Data are expressed as mean ± SEM. (C) Close ups of the uteri extracted from mice treated with QD administration of vehicle and AZD4547 at the dose of 25 mg/kg p.o for 20 days from 2 weeks after endometriosis induction. (D) Typical estrous cycle of mice following endometriosis induction and QD treatment with vehicle and AZD4547 p.o from 2 weeks after endometriosis induction for 20 days. (E) Typical H&E staining of endometriotic lesions established in the mice treated QD with vehicle and AZD4547 p.o from 2 weeks after endometriosis induction for 20 days. Scale bars and magnification are respectively 1,000 μm and 1×. In the images: pw: peritoneal wall; L: lumen; om: omental adhesion; g: glandular structure with glandular epithelium; le: luminal epithelium; s: stroma; l: leukocytes

The transplanted syngeneic uterine fragments were left to develop for 2 weeks post‐surgery before treatment was administered. Lesions developed in all groups having the appearance of large cysts filled with fluid, clumped together and with omental adhesion. Reduction in the lesion size was noticed in the group treated with AZD4547 at the highest dose compared to the others (Figure 7A). As observed when the compound was administered from the day of surgery, no significant weight gain or loss was experienced by the animals, confirming the tolerability of the drug (Figure 7B). Uteri showed no alterations between the groups, although the macroscopic appearance was different according to the stage of the estrous cycle (uteri at proestrus appeared more swollen compared to the others in other stages of the cycle), as observed when AZD4547 was administered from the day of surgery (Figure 7C).

Daily examination of vaginal cytology confirmed the lack of disruption of the estrous cycle induced by AZD4547, as can be seen by the regularity of the 4‐5 day cycle in all groups, confirming what was observed when the drug was administered from the day of endometriosis induction (Figure 7D). H&E staining confirmed the viable architecture of the lesions, which again showed luminal epithelium, stroma with glandular structures, lumen filled with leukocytes, and omental adhesion in all groups with reduction of the lesion size in the 25 mg/kg group compared to the other groups (Figure 7E).

**FIGURE 8:**
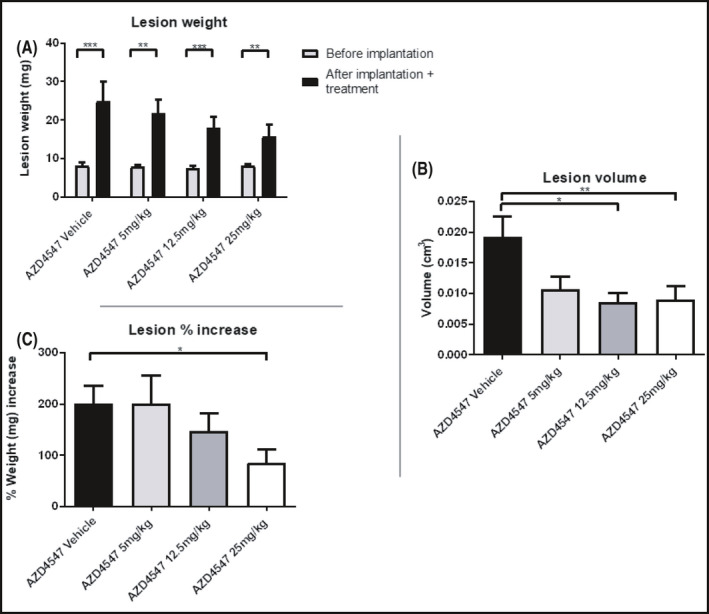
(A) Weight of the uterine fragments before being transplanted and after sacrifice following 20‐day QD treatment with vehicle, AZD4547 at the doses of 5, 12.5, and 25 mg/kg p.o starting from 2 weeks after endometriosis induction. (B) Final lesion volume (cm^3^). (C) Percentage increase in lesion weight. Data are expressed as mean ± SEM. Animal number per group *n* = 6, lesion number per group, *n* = 18, with the exception of the groups treated with the vehicle of AZD4547, whose lesion number was *n* = 15

The increase in the weight of the lesions from the day of implantation to the day of harvest was statistically significant in all the groups (***p < 0.001 in the groups treated with AZD4547 vehicle and 12.5 mg/kg, **p < 0.01 in the groups treated with 5 and 25 mg/kg, Figure 8A). Lesion volume decreased from vehicle group compared to the groups treated with AZD4547 at 12.5 (*p < 0.05) and 25 mg/kg (**p < 0.01) (Figure 8B **)**. Compared to its vehicle, AZD4547 at the dose of 5 mg/kg was responsible for 44.3% volume reduction, the dose of 12.5 mg/kg for 55.6% and the dose of 25 mg/kg for 53.6%.

Lesion weight (expressed as a percentage increase in weight of the original fragment implanted, Figure 8C) was significantly higher in the AZD4547 vehicle group (*p < 0.05) compared to the 25 mg/kg group. Compared to its vehicle, AZD4547 at the dose of 5 mg/kg was responsible for a lesion weight regression of 0.32%, the dose of 12.5 mg/kg of 27.1% and the dose of 25 mg/kg of 57.7%. Percentage of lesions filled with fluid decreased from the vehicle (100%) to the drug‐treated groups (67% in the 5 mg/kg group, 78% in the 12.5 and 25 mg/kg groups).

### 3.5 ENG administered from S2

**FIGURE 9:**
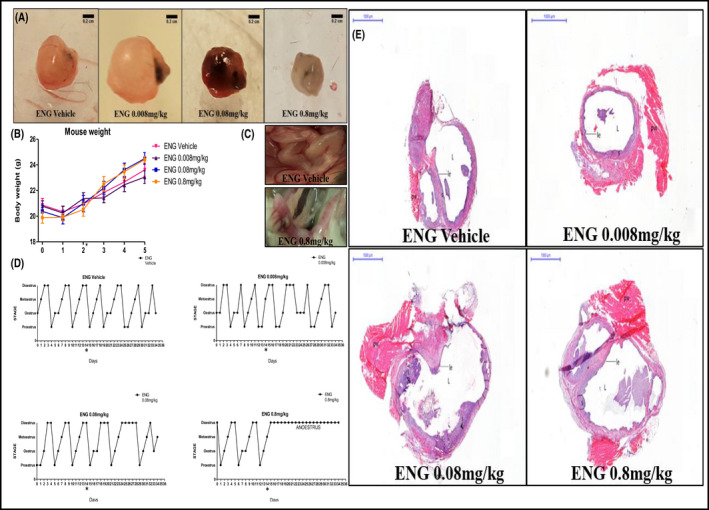
(A) Close ups of the lesions extracted from mice treated QD with administration of vehicle and ENG at the doses of 0.008, 0.08, and 0.8 mg/kg s.c. for 20 days from 2 weeks after endometriosis induction (the black nonabsorbable sutures used to attach the uterine fragments to the peritoneal wall were still visible). Scale bars are 0.2 cm. (B) Mice weight (g) fluctuations at Week 1, 2, 3, 4, and 5 post‐surgery and treatment. The symbol * by Week 2 indicates the beginning of the treatment. Data are expressed as mean ± SEM. (C) Close ups of the uteri extracted from mice treated with QD administration of vehicle and ENG at the dose of 0.8 mg/kg s.c. for 20 days from 2 weeks after endometriosis induction. As observed when ENG was administered from the day of surgery, the uterus from the mouse treated with the highest dose of the drug appeared hyperemic with vascular engorgement. (D) Typical estrous cycle of mice following endometriosis induction and QD treatment with vehicle and ENG s.c. from 2 weeks after endometriosis induction for 20 days. (E) Typical H&E staining of endometriotic lesions established in the mice treated QD with vehicle and ENG s.c. from 2 weeks after endometriosis induction for 20 days. Scale bars and magnification are respectively 1,000 μm and 1×. In the images: pw: peritoneal wall; L: lumen; om: omental adhesion; g: glandular structure with glandular epithelium; le: luminal epithelium; s: stroma; l: leukocytes

When ENG was administered from 2 weeks after endometriosis induction, the developed lesions showed no macroscopic difference in size or appearance between the groups. Development of blood filled cysts was observed in one out of the six mice treated with the dose of 0.08 mg/kg (Figure 9A). As observed when the compound was administered from the day of surgery, no significant weight gain or loss was experienced by the animals, confirming the tolerability of the drug (Figure 9B). However, as seen when the treatment with ENG was started from the day of endometriosis induction, uteri appeared increasingly hyperemic in the ENG treated groups compared to the vehicle group (the hyperemia seemed to increase with the increasing of the dose of the drug [Figure 9C]).

When ENG was administered from 2 weeks after surgery, the vehicle and the lowest dose (0.008 mg/kg) groups similarly showed non impairment of the 4‐5 day cycle; temporary interruption followed by recovery of the cycling regularity was observed in the 0.08 mg/kg treatment groups; whereas the doses 0.8 mg/kg caused complete interruption of the cycle and the establishment of the anestrus, with observation of mucus and thickening in the vaginal smear (as observed when the drug was administered from the day of endometriosis induction, Figure 9D). No histologic difference was observed in the lesions between all groups (H&E staining confirmed the lesions well developed architecture in all groups, Figure 9E).

**FIGURE 10:**
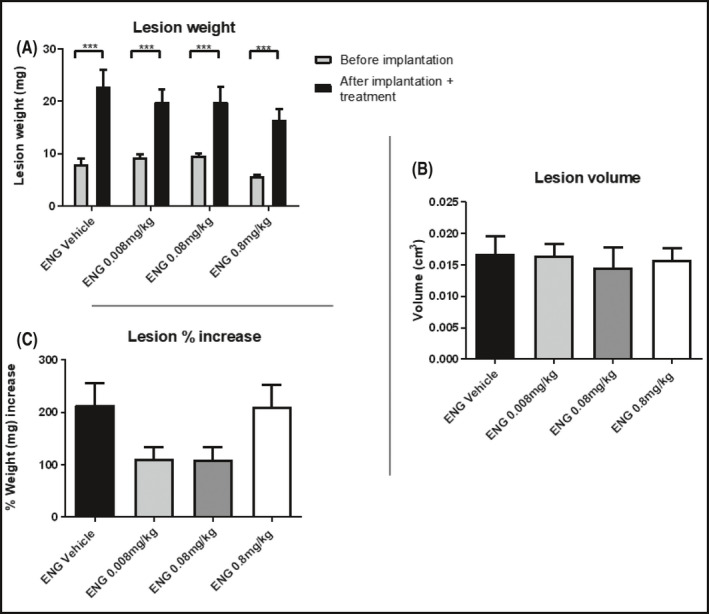
(A) Weight of the uterine fragments before being transplanted and after sacrifice following 20‐day QD treatment with vehicle and ENG at the doses of 0.008. 0.08, and 0.8 mg/kg s.c. starting from 2 weeks after endometriosis induction. (B) Final lesion volume (cm^3^). (C) Percentage increase in lesion weight. Data are expressed as mean ± SEM. Animal number per group *n* = 6, lesion number per group, *n* = 18, with the exception of the groups treated with ENG at the dose of 0.8 mg/kg, whose lesion number was *n* = 17

The increase in the weight of the lesions from the day of implantation to the day of harvest was statistically significant in all the groups (***p < 0.001, Figure 10A). Lesion volumes in the ENG treated groups were very similar and not considerably lower than the vehicle (Figure 10B). Compared to its vehicle, ENG at the dose of 0.008 mg/kg reduced the volume only by 2%, the dose of 0.08 mg/kg by 13.2% and the dose of 0.8 mg/kg by 6%.

Lesion weight (expressed as a percentage increase in weight of the original fragment implanted, Figure 10C) was not significantly higher in the ENG vehicle group compared to the 0.008 and 0.08 mg/kg group, and very similar to the 0.8 mg/kg group. Compared to its vehicle, ENG at the dose of 0.008 mg/kg induced lesion weight regression of 47.6%, the dose of 0.08 mg/kg of 48.9%, and the dose of 0.8 mg/kg of 6.7% only (Table S5). Percentage of lesions filled with fluid decreased from the vehicle (100%) to the drug‐treated groups (78% in the 0.008 mg/kg group, 61% in the 0.08 mg/kg group, and 71% in the 0.8 mg/kg group).

## 4 DISCUSSION AND CONCLUSION

The results obtained from our animal model showed that the transplant of endometrial tissue into the peritoneum to simulate the retrograde menstruation process (the most widely accepted theory explaining the aetiology of endometriosis),^1^ was the only necessary requirement for the establishment of endometriotic lesions. In our model, surgery was performed when donor mice were in proestrus and the level of endogenous estrogen was high.^14^

In 2004, Fortin et al.^21^ demonstrated estrogen supplementation was not essential for the establishment of endometriosis as the estradiol and ERα present in the uterine fragments transplanted in the recipient mice was sufficient to induce the lesion implantation. The essential role of estrogen for the maintenance of the disease but not for the initiation of the disease was proved in other studies conducted on mice and monkeys.^22^, ^23^, ^24^ In 2018, Kiani et al. demonstrated the maintenance of the endogenous estrogen levels in mice as the only necessary requirement for the development of endometriosis, with no significant difference between the stages of the estrous cycle.^25^

In our study, the therapeutic efficacy of the novel drug AZD4547 and ENG was evaluated through their administration for 20 days starting from the day of endometriosis induction, to represent a proxy for prophylactic treatment, and 2 weeks after surgery, when lesions were already established.^12^ ENG represents a first line progestin treatment which, compared to the previous generation progestins, displays the same anti‐estrogenic activity but less androgenic and anabolic side effects because of its lower binding to the androgen, mineralocorticoid, glucocorticoid receptors and more selectivity towards PR.^26^

Vehicle and treatment with AZD4547 from the day of surgery and 2 weeks after surgery were well tolerated by the animals: no remarkable weight gain or loss, abnormal behavioral changes, or alterations in the estrous cycle were observed. Gross pathology examination demonstrated fluid‐filled lesion development in all the groups of mice treated with the vehicle (PEG400) and AZD4547 at the three different doses starting either from the day of surgery or 2 weeks after; however, in both cases, the tissue implants in the mice treated with 25 mg/kg showed the greatest macroscopic size and lesion reduction, as confirmed by H&E staining, analysis of lesion weight, volume, percentage increase from the day of implantation and of lesions filled with fluid. Highest efficacy was observed when AZD4547 was administered from the day of endometriosis induction. AZD4547 anti‐estrogenic‐like activity was demonstrated by the regression of the endometriotic lesions and reduction in FGFR2 expression in a dose‐dependent manner, confirming what was observed in preclinical tumor growth inhibition studies. The doses of 25 and 12.5 mg/kg proved to be effective in non‐small cell lung cancer (NSCLC) FGFR1‐amplified patient‐derived tumor xenograft (PDTX) models.^27^ In an in vivo endometrial xenograft model, tumors reached a final volume of approximately 1,000 mm^3^ in the vehicle group, whereas they were reduced to 360 mm^3^ in the group treated with AZD4547 at a dose of 10 mg/kg for 15 days by oral gavage. At a dose of 30 mg/kg, the tumor establishment was slower and, in some cases, with episodes of complete regression within a week.^10^ In another in vivo mouse model, mice received inoculation of mouse mammary tumor 4 T1 cells and 7 days after, they were treated by intraperitoneal injection with AZD4547 at the dose of 5 mg/kg for 20 days.^17^

Similarly administration of vehicle and ENG for 20 days from the day of surgery and 2 weeks after surgery induced no remarkable change in the weight or abnormal behavior in the animals. When ENG was administered from the day of surgery, therapeutic efficacy was displayed at all doses. Gross pathology examination of the lesions showed well‐developed fluid‐filled endometriotic lesions in the vehicle group, smaller and not always fluid‐filled lesions in the 0.008 and 0.08 mg/kg groups and no fluid‐filled lesion formation, but just the uterine pieces attached to the peritoneal wall in the 0.8 mg/kg group. Analysis of lesion weight, volume, percentage increase, and lesions filled with fluid confirmed ENG efficacy in counteracting endometriotic growth at all the three doses tested; however, 0.8 mg/kg proved to be the most effective dose. Uteri appeared swollen, well vascularized in all the groups and more hyperemic with the increasing doses of ENG. Several studies report enlargement, increase in the number, and fragility of the blood vessels in the endometrium of women subjected to long term treatment with progestins due to changes in ERα and FGFR expression.^28^, ^29^, ^30^ These abnormalities in the angiogenesis process could explain the unwanted uterine bleeding associated with long‐term use of progestins, which decreases the patient compliance and compromises their adherence. Similarly, studies conducted on guinea pigs treated with ENG confirmed morphological changes in the endometrial lining of the uterus with histological evidence of inflammation, hemorrhage, decrease in the number of endometrial glands, and hyperemic uteri.^31^

ENG exerted anti‐estrogenic‐like activity regressing endometriotic lesions in a dose‐dependent manner by attenuating both FGFR1 and FGFR2 expressions. FGFRs are co‐localized in arteries, endometrial glands, and luminal epithelium,^32^ which synergistically promote cell proliferation, tumor growth, and angiogenesis.^33^ Despite displaying the highest therapeutic efficacy, the 0.8 mg/kg dose of ENG was associated with anti‐ovulatory activity regardless of treatment administration times, as demonstrated by thicker mucus in the cervical smear, complete interruption of the estrous cycle, and establishment of the anestrous stage. Histological analysis of the ovaries showed atrophic follicles in the 0.8 mg/kg group, which were not observed in the lower dose treatment groups (Figure S3). When ENG was administered from 2 weeks after surgery, gross pathology examination and H&E staining showed well‐developed fluid‐filled endometriotic lesions in all the groups, with no macroscopic differences or size reduction between the groups. Lesions developed in the vehicle group were not statistically different in weight, volume, and percentage of increase compared to the ENG‐treated groups.

When administered systemically from the day of endometriosis induction, ENG was more effective than AZD4547 with respect to the reduction of percentage of fluid‐filled cysts at every dose (Table S6), which could be attributed to differential expression of FGFR1 transcripts. However, AZD4547 at the dose of 25 mg/kg matched the efficacy shown by every dose of ENG towards percentage lesion weight reduction. ENG was responsible for inducing hyperemia in the uteri, thickening, and mucus in the cervical smear and complete or partial (respectively for 0.8 mg/kg and 0.08 mg/kg) interruption of the estrous cycle and reduced fertility. Conversely, AZD4547 did not cause uterine hyperemia, estrous cycle disturbance, or interruption at any dose. In women, this would be desirable as it would not impair fertility. When administered 2 weeks after endometriosis induction, hyperemia of the uteri and cycle disruption were again observed in the ENG‐treated groups but not in the AZD4547 groups. In contrast to ENG, which was reported in several studies to induce endometrial upregulation of VEGF and Ang‐2 (the two main mediators of endometrial angiogenesis, vascular density and remodeling, including branching and enlargement ^34^, ^35^, ^36^, ^37^), AZD4547, through the blockage of the FGFR pathway, is responsible for the inhibition of endothelial cell proliferation, migration, differentiation, survival,^18^, ^38^ and VEGFR and platelet‐derived growth factor receptor (PDGFR) signaling pathways.^5^, ^6^

H&E staining showed well‐developed lesions in all the groups treated with both AZD4547 and ENG; however, the lumen size of the lesions in the mice treated 2 weeks after surgery with AZD4547 at 25 mg/kg appeared smaller compared to the groups treated with any dosage of ENG. ENG at 0.08 mg/kg was more effective than any dose of AZD4547 with respect to the reduction of percentage of fluid‐filled cysts (Table S7 **)**; however, AZD4547 displayed higher percentage lesion weight reduction at the dose of 25 mg/kg and higher volume regression (shown as a percentage change from the vehicle) at every dose compared to ENG, confirming the highest dose of AZD4547 to be more effective than ENG also when administered 2 weeks after endometriosis induction (Table S5).

The inhibitory effect of AZD4547 on the establishment and progression of the endometriotic lesions, without impairing estrous cycle regularity, suggests clinical usefulness of this novel drug for short‐ or long‐term prophylactic or management therapies. The administration of ENG, instead, proved to be effective only when administered from the day of surgery and not when the lesions were already developed. Moreover, ENG was associated with the unwanted impairment of fertility via ovarian atrophy and estrous cycle interruption (Figure S3).

In conclusion, our results confirm that targeting FGFR signaling could form the basis of a new strategy for endometriosis therapy. Based on the promising results obtained by measurement of lesion weight, volume, and regularity of the estrous cycle, AZD4547 is worth further investigation as a potential treatment for endometriosis, widening the spectrum of the therapies currently available.

## CONFLICT OF INTEREST

The authors declare no conflicts of interest.

## Supporting information

Supplementary Material

## Data Availability

The data that supports the findings of this study are available in the supplementary material of this article.
