# Supplementary material for: In vivo effects of AZD4547, a novel fibroblast growth factor receptor inhibitor, in a mouse model of endometriosis
Source: Pharmacol Res Perspect. 2021 Apr 3;9(2):e00759. doi: 10.1002/prp2.759 (PMC8019068; doi:10.1002/prp2.759)
Supplement: Supplementary file 1 — Supplementary Material [file PRP2-9-e00759-s001.docx]

**Supplementary data**

***Supplementary Table 1****: Primers pairs used for qRT-PCR*

| **Primer** | **Sequence** | **Amplicon length** | **Accession Number** |
| --- | --- | --- | --- |
| Β-actin (F)  Β-actin (R) | 5’-GATGTATGAAGGCTTTGGTC-3’  5’-TGTGCACTTTTATTGGTCTC- 3’ | 96 | NM_007393.5 |
| GAPDH (F)  GAPDH (R) | 5’-TGTTCGTCATGGGTGTGAAC-3’  5’-TGTGGTCATGAGTCCTTCCA-3’ | 143 | NM_002046.5 |
| ERα (F)  ERα (R) | 5’-GCTCCTAACTTGCTCCTGGAC-3’  5’-CAGCAACATGTCAAAGATCTCC-3’ | 75 | NM_001302531.1 |
| PR A/B (F)  PR A/B (R) | 5’-CTAAATGAGCAGAGGATGA-3’  5’-CTTCAAACTGGCTTTGACTC-3’ | 178 | NM_008829.2 |
| FGFR1 (F)  FGFR1 (R) | 5’-TATGTCCAGATCCTGAAGAC-3’  5’-GAGAGTCCGATAGAGTTACC-3’ | 131 | NM_001079908.2 |
| FGFR2 (F)  FGFR2 (R) | 5’-CAGTGGGAATCGATAAAGAC-3’  5’-TCAACTATGACGTAGAGAGG-3’ | 196 | NM_201601.2 |
| FRS2 (F)  FRS2 (R) | 5’-ACCATCGGAACAAGTTTAAG-3’  5’-CAGAGGTAGTGCCATTTTAC-3’ | 136 | NM_177798.3 |

***Supplementary Table 2****: Endometriotic lesions take rate (%) in mice treated with AZD4547 and ENG for 20 days, starting either from the day of endometriosis induction (S0) or two weeks after endometriosis induction (S2) (animal number per group n=6, lesion number per group, n=18, with the exception of the groups treated with the vehicle of AZD4547 from S2, whose lesion number was n=15, and the groups treated with ENG at the dose of 0.8mg/kg, whose lesion number was n=17).*

| **Treatment group** | **Treatment dose** | **Take rate**  **(%)** |
| --- | --- | --- |
| AZD4547 S0 | Vehicle | 94 |
|  | 5mg/kg | 100 |
|  | 12.5mg/kg | 83 |
|  | 25mg/kg | 72 |
| AZD4547 S2 | Vehicle | 100 |
|  | 5mg/kg | 89 |
|  | 12.5mg/kg | 89 |
|  | 25mg/kg | 89 |
| ENG S0 | Vehicle | 94 |
|  | 0.008mg/kg | 61 |
|  | 0.08mg/kg | 72 |
|  | 0.8mg/kg | 61 |
| ENG S2 | Vehicle | 100 |
|  | 0.008mg/kg | 94 |
|  | 0.08mg/kg | 94 |
|  | 0.8mg/kg | 88 |

***Supplementary Figure 1****: Individual endometriotic lesion weight (mg) in each animal according to the stage of the cycle at S0 and treatment received with* *AZD4547 and ENG starting either from S0* ***(A, C)*** *or two weeks after (S2)****(B, D).***

***Supplementary Table 3****: Number of animals in each stage of the cycle at the moment of sacrifice (S1) for each treatment group.*

| **Treatment group** | **Treatment dose** | **Proestrus** | **Oestrus** | **Metoestrus** | **Dioestrus** | **Anoestrus** |
| --- | --- | --- | --- | --- | --- | --- |
| AZD4547 S0 | Vehicle |  |  | 1 | 5 |  |
|  | 5mg/kg | 1 | 1 | 2 | 2 |  |
|  | 12.5mg/kg | 3 | 1 |  | 2 |  |
|  | 25mg/kg | 2 | 1 | 1 | 2 |  |
| AZD4547 S2 | Vehicle | 3 |  | 1 | 2 |  |
|  | 5mg/kg | 3 | 1 | 1 | 1 |  |
|  | 12.5mg/kg | 2 |  |  | 4 |  |
|  | 25mg/kg | 4 |  | 1 | 1 |  |
| ENG S0 | Vehicle | 1 |  | 1 | 4 |  |
|  | 0.008mg/kg | 1 | 1 | 2 | 2 |  |
|  | 0.08mg/kg |  |  | 1 | 5 |  |
|  | 0.8mg/kg |  |  |  |  | 6 |
| ENG S2 | Vehicle | 2 | 2 | 1 | 1 |  |
|  | 0.008mg/kg | 2 | 1 | 2 | 1 |  |
|  | 0.08mg/kg |  | 1 | 1 | 4 |  |
|  | 0.8mg/kg |  |  |  |  | 6 |

***Supplementary Figure 2****: Individual endometriotic lesion weight (mg) in each animal according to the stage of the cycle at the moment of sacrifice (S1) and treatment received with* *AZD4547 and ENG starting from S0* ***(A, C)*** *and two weeks after (S2)****(B, D).***

***Supplementary Table 4:*** *Lesion percentage increase and volume expressed as a percentage change from the vehicle (expressed as mean ± SEM of the lesion percentage increase and volume values). Lesions were extracted from mice treated by oral gavage once a day, with vehicle of AZD4547, AZD4547 at the doses of 5mg/kg, 12.5mg/kg, 25mg/kg, and, by subcutaneous injection, once a day, with vehicle of ENG, ENG at the doses of 0.008mg/kg, 0.08mg/kg, 0.8mg/kg for 20 days, starting from the day of endometriosis induction. Data are expressed as mean ± SEM (animal number per group n=6, lesion number per group, n=18). Significances:* ***A:*** **P<0.05 when 5mg/kg AZD4547 was compared to 0.008mg/kg ENG. *P<0.05 when 5mg/kg AZD4547 was compared to 0.08mg/kg ENG. *P<0.01 when 5mg/kg AZD4547 was compared to 0.8mg/kg ENG.* ***B:*** **P<0.05 when 12.5mg/kg AZD4547 was compared to 0.8mg/kg ENG.* ***C:*** **P<0.05 when AZD4547 vehicle was compared to ENG-vehicle.* ***D:*** ****P<0.001 when 5mg/kg AZD4547 was compared to 0.008mg/kg ENG. **P<0.01 when 5mg/kg AZD4547 was compared to 0.08mg/kg ENG.* ***E:*** ***P<0.01 when 12.5mg/kg AZD4547 was compared to 0.008mg/kg ENG. *P<0.05 when 12.5mg/kg AZD4547 was compared to 0.08mg/kg ENG.* ***F:*** **** P<0.001 when 25mg/kg AZD4547 was compared to 0.008mg/kg ENG. P<0.001 when 25mg/kg AZD4547 was compared to 0.08mg/kg ENG.*

| Parameter | | AZD4547  dose | Mean  ±  SEM | ENG  dose | Mean  ±  SEM | Significances |
| --- | --- | --- | --- | --- | --- | --- |
|  | Vehicle | | **147.8**  **±**  **2.6** | Vehicle | **154.9**  **±**  **27.2** |  |
| % change from vehicle: lesion percentage increase | 5mg/kg | | **11.4**  **±**  **29.2** | 0.008mg/kg | **-76.6**  **±**  **11.7** | **A** |
|  | 12.5mg/kg | | **-12.2**  **±**  **19.2** | 0.08mg/kg | **-77.1**  **±**  **10.7** | **B** |
|  | 25mg/kg | | **-83.3**  **±**  **9.3** | 0.8mg/kg | **-89.2**  **±**  **5.2** |  |
|  | Vehicle | | **0.028**  **±**  **0.008** | Vehicle | **0.008**  **±**  **0.001** | **C** |
| % change from vehicle: lesion volume | 5mg/kg | | **-63.3**  **±**  **9.4** | 0.008mg/kg | **-10.6**  **±**  **12.2** | **D** |
|  | 12.5mg/kg | | **-62.5**  **±**  **8.6** | 0.08mg/kg | **-12.7**  **±**  **17.3** | **E** |
|  | 25mg/kg | | **-73.2**  **±**  **4.4** | 0.8mg/kg | **-50.0**  **±**  **6.0** | **F** |

***Supplementary Table 5:*** *Lesion percentage increase and volume expressed as a percentage change from the vehicle (expressed as mean ± SEM of the lesion percentage increase and volume values). Lesions were extracted from mice treated by oral gavage once a day, with vehicle of AZD4547, AZD4547 at the doses of 5mg/kg, 12.5mg/kg, 25mg/kg, and, by subcutaneous injection, once a day, with vehicle of ENG, ENG at the doses of 0.008mg/kg, 0.08mg/kg, 0.8mg/kg for 20 days, starting from 2 weeks after endometriosis induction. Data are expressed as mean ± SEM (animal number per group n=6, lesion number per group, n=18 with the exception of the groups treated with the vehicle of AZD4547, whose lesion number was n=15, and the groups treated with ENG at the dose of 0.8mg/kg, whose lesion number was n=17). Significances:* ***A:*** ***P<0.01 when 12.5mg/kg AZD4547 was compared to 0.008mg/kg and 0.8mg/kg ENG.* ***B:*** ***P<0.01 when 25mg/kg AZD4547 was compared to 0.008mg/kg and 0.8mg/kg ENG.*

| Parameter | AZD4547  dose | Mean  ±  SEM | ENG  dose | Mean  ±  SEM | Significances |
| --- | --- | --- | --- | --- | --- |
|  | Vehicle | **201.4**  **±**  **35.0** | Vehicle | **214.3**  **±**  **40.8** |  |
| % change from vehicle: lesion percentage increase | 5mg/kg | **-0.3**  **±**  **27.5** | 0.008mg/kg | **-47.7**  **±**  **10** |  |
|  | 12.5mg/kg | **-27.1**  **±**  **17.2** | 0.08mg/kg | **-48.9**  **±**  **10.9** |  |
|  | 25mg/kg | **-57.6**  **±**  **13.2** | 0.8mg/kg | **-6.7**  **±**  **19.2** |  |
|  | Vehicle | **0.019**  **±**  **0.003** | Vehicle | **0.017**  **±**  **0.003** |  |
| % change from vehicle: | 5mg/kg | **-44.3**  **±**  **10.1** | 0.008mg/kg | **-1.8**  **±**  **11.4** |  |
| lesion volume | 12.5mg/kg | **-55.6**  **±**  **8.1** | 0.08mg/kg | **-13.2**  **±**  **18.9** | **A** |
|  | 25mg/kg | **-53.6**  **±**  **11.7** | 0.8mg/kg | **-6.0**  **±**  **11.1** | **B** |

***Supplementary Figure 3:*** *Typical H&E staining of ovaries from mice treated QD from the day of endometriosis induction with vehicle and with ENG s.c. at the dose of* *0.008, 0.08 and 0.8 mg/kg. Scale bars and magnification are respectively 500µm and 3x and 4x in the ENG 0.8mg/kg picture.* ***Of****: ovarian follicle;* ***s****: stroma;* ***cl****: corpus luteum;* ***af****: atrophic follicle.*


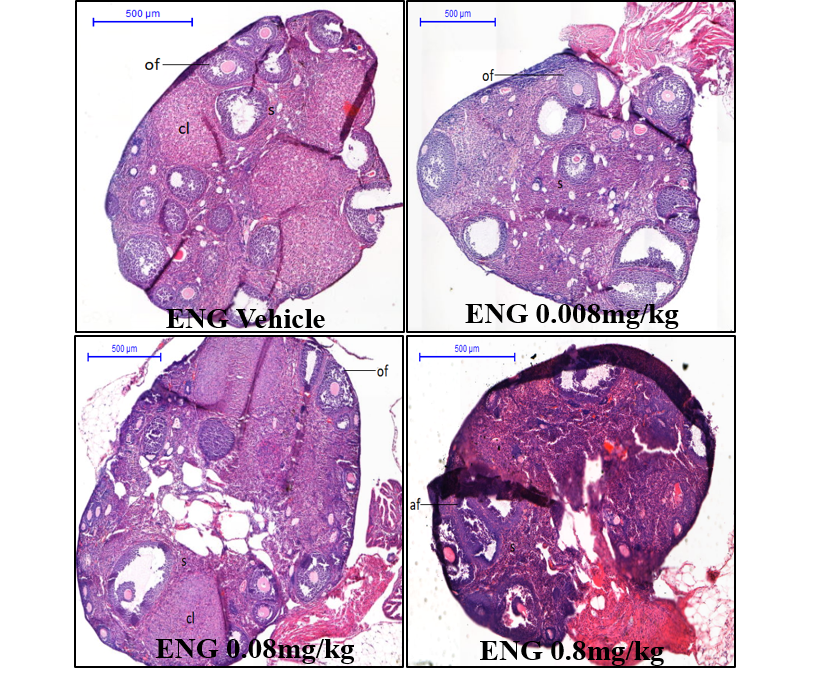


***Supplementary Table 6:*** *Comparison of percentage lesion filled with fluid from mice treated by oral gavage once a day, with vehicle of AZD4547, AZD4547 at the doses of 5mg/kg, 12.5mg/kg, 25mg/kg, and, by subcutaneous injection once a day with vehicle of ENG, ENG at the doses of 0.008mg/kg, 0.08mg/kg, 0.8mg/kg for 20 days, starting from the day of endometriosis induction (animal number per group n=6, lesion number per group, n=18).*

| Parameter | AZD4547  dose | % lesions  with fluid | ENG  dose | % lesions  with fluid |
| --- | --- | --- | --- | --- |
|  | Vehicle | 89 | Vehicle | 94 |
| % lesions | 5mg/kg | 78 | 0.008mg/kg | 50 |
| filled with fluid | 12.5mg/kg | 78 | 0.08mg/kg | 44 |
|  | 25mg/kg | 78 | 0.8mg/kg | 0 |

***Supplementary Table 7:*** *Comparison of percentage lesion filled with fluid from mice treated by oral gavage once a day, with vehicle of AZD4547, AZD4547 at the doses of 5mg/kg, 12.5mg/kg, 25mg/kg, and, by subcutaneous injection once a day with vehicle of ENG, ENG at the doses of 0.008mg/kg, 0.08mg/kg, 0.8mg/kg for 20 days, starting from 2 weeks after endometriosis induction (animal number per group n=6, lesion number per group, n=18).*

| Parameter | AZD4547  dose | % lesions  with fluid | | ENG  dose | % lesions  with fluid |
| --- | --- | --- | --- | --- | --- |
|  | Vehicle | 100 | | Vehicle | 100.0 |
| % lesions | 5mg/kg | | 67 | 0.008mg/kg | 73 |
| Filled with fluid | 12.5mg/kg | | 72 | 0.08mg/kg | 61 |
|  | 25mg/kg | | 78 | 0.8mg/kg | 71 |
